# Outcome and Determinants of Outcome of COVID-19 Infection Among Hemodialysis Patients: Findings From a National Dialysis Network Program in India

**DOI:** 10.1016/j.ekir.2021.03.003

**Published:** 2021-03-15

**Authors:** Tom Jose Kakkanattu, Suresh Sankarasubbaiyan, Ashok K. Yadav, Monica Kundu, Mallikarjuna Gowda BG, Vivek Kumar, Kamal Shah, Vivekanand Jha

**Affiliations:** 1 Department of Nephrology, Postgraduate Institute of Medical Institute Education and Research, Chandigarh, India; 2 NephroPlus Dialysis Network, Hyderabad, India; 3 Department of Experimental Medicine and Biotechnology, Postgraduate Institute of Medical Institute Education and Research, Chandigarh, India; 4 George Institute for Global Health, UNSW, New Delhi, India; 5 School of Public Health, Imperial College, London, UK; 6 Manipal Academy of Higher Education, Manipal, India

The severe acute respiratory syndrome coronavirus 2 (SARS-CoV-2), the causative agent of the coronavirus disease 2019 (COVID-19) pandemic, has infected nearly 124 million people and caused more than 275,000 deaths worldwide. Patients with pre-existing conditions including those with chronic kidney disease are at increased risk of adverse outcomes because of this infection. In the United States, the risk of COVID-19 infection was 3.5 times greater among Medicare end-stage renal disease beneficiaries compared to all fee-for-service beneficiaries. Dialysis patients, already at high risk for a variety of complications, in particular cardiovascular disease, are also at an increased risk of adverse outcomes secondary to COVID-19 because of age, comorbidities such as diabetes, hypertension, and the need for multiple hospital contacts for dialysis. Most reports of COVID-19 in dialysis patients are from the United States and Europe, and the impact of the pandemic on dialysis patients in the developing world is lacking.^1^, ^2^, ^3^, ^4^ Initial reports from India highlighted the large-scale hardships faced by the inability of the patient on dialysis to access treatment during the prolonged period of nationwide lockdown leading to missed treatments and dropouts.^5^^,^^6^ In this article, we report the outcomes of patients diagnosed with COVID-19 in-center in a large hemodialysis network in India.

## Results

Of a total 14,573 patients who received dialysis in the network centers during the entire study period, 1279 subjects were found to be positive for SARS-CoV-2. Table 1 describes the demographic characteristics and comorbidities for all subjects. The mean age was 53.63 years, and male patients were predominant (72.2%). Patients had been on dialysis for 590 ± 725 days before diagnosis. The most common comorbidities were hypertension in 39.85%, diabetes in 20.31%, and heart disease in 6.57%.

**Table 1:** Description of COVID-19–positive hemodialysis subjects

	Total (N = 1279)	Males n = 923 (72.17%)	Females n = 356 (27.83%)
Age, years	53.63 ± 13.30	54.14 ± 13.30	52.27 ± 13.22
<30	67 (5.50)	45 (5.14)	22 (6.60)
30–55	597(49.50)	427 (48.74)	170 (51.40)
>55	543 (45.00)	404 (46.12)	139 (42.00)
Duration of hospital stay, days	11.95 ± 7.00	11.60 ± 6.62	12.88 ± 7.86
Range	1 to 39	1 to 39	1 to 39
Dialysis vintage, days	590 ± 725	569 ± 716	648 ± 747
Range	1 to 4032	1 to 4032	1 to 3554
Reason for COVID-19 testing			
Symptom-based	805 (62.94)	583 (63.16)	222 (62.36)
Exposed in unit	86 (6.72)	67 (7.25)	19 (5.33)
Contact in neighborhood/home	20 (1.56)	15 (1.63)	5 (1.40)
Travel history	4 (0.31)	4 (0.43)	—
Unknown	364 (28.46)	254 (27.52)	110 (30.90)
Outcome			
Discharged	969 (75.76)	693 (75.08)	276 (77.53)
Expired	293 (22.91)	219 (23.73)	74 (20.79)
Treating at home	17 (1.33)	11 (1.19)	6 (1.69)
Referred from another facility			
No	902 (70.52)	650 (70.42)	252 (70.79)
Yes	377 (29.48)	273 (29.58)	104 (29.21)
Payment type			
Out of pocket	210 (22.15)	150 (21.25)	60 (24.79)
Public insurance	677 (71.41)	514 (72.80)	163 (67.36)
Private insurance	61 (6.43)	42 (5.95)	19 (7.85)
Regular exercise	148 (12.63)	114 (13.43)	34 (10.53)
Tobacco use^a^	128 (10.95)	118 (13.93)	10 (3.11)
Hypertension	467 (39.85)	335 (39.46)	132 (40.87)
Diabetes	238 (20.31)	176 (20.73)	62 (19.20)
Dyslipidemia	18 (1.54)	16 (1.88)	2 (0.62)
Heart disease	77 (6.57)	58 (6.83)	19 (5.88)

Data presented as mean ± SD and n (%).

a Includes smoking as well as smokeless (chewable) tobacco

The main indications for testing for SARS-CoV-2 were the presence of symptoms in 805 (63%), and contact with SARS-CoV-2–positive patients in hospital in 86 (6.72%). A total of 377 (29.48%) patients were referred from another dialysis facility after receiving a COVID-19 diagnosis because the referring unit did not have the facility to dialyze these patients. A majority of the patients (1262, 98.67%) were hospitalized after being diagnosed with COVID-19. The duration of hospital stay was 11.95 ± 7 days. The distribution of variables in subjects who survived and who expired are shown in Table 2. Of the COVID-19–positive population, 293 (22.91%) expired. During the same time, there were 2560 deaths among the 13,294 COVID-19–negative population in the network, giving a mortality rate of 19.26%. In comparison, this death rate during the same period in the previous year (2019) was 15%.

**Table 2:** Variables among COVID-19–positive patients who died and survived

	Died n = 293 (22.91)	Survived n = 986 (77.09)
Sex		
Female	74 (25.26)	282 (28.60)
Male	219 (74.74)	704 (71.40)
Referred from another facility		
No	239 (81.57)	663 (67.24)
Yes	54 (18.43)	323 (32.76)
Smoker/tobacco user	24 (8.70)	104 (11.65)
Exercise	36 (12.90)	112 (12.54)
Diabetes	75 (26.88)	163 (18.25)
Dyslipidemia	6 (2.15)	12 (1.34)
Heart disease	27 (9.68)	50 (5.60)
Hypertension	140 (50.18)	327 (36.62)
Age, years	56.51 ± 12.74	52.73 ± 13.35
Dialysis duration, days	786 ± 826	531 ± 681
Range	1 to 3481	1 to 4032
Length of hospitalization, days	8.33 ± 7.10	13.1 ± 6.52
Range	1 to 35	1 to 39

Data presented as n (%) and mean ± SD.

Compared to those who survived the illness, the COVID-19–positive patients who died were older (ages 56.51 ± 12.74 years vs. 52.73 ± 13.35 years, *P* < 0.001), and had longer dialysis vintage (786 ± 826 days vs. 531 ± 681 days, *P* < 0.001). Mortality in subjects older than 55 years was more than three-fold higher as compared to subjects younger than 30 years (*P* = 0.014). Diabetes (odds ratio [OR]: 1.65; 95% confidence interval [CI]: 1.20 to 2.25; *P* = 0.002), hypertension (OR: 1.74; 95% CI: 1.33 to 2.29; *P* < 0.001), heart disease (OR: 1.81; 95% CI: 1.11 to 2.95; *P* = 0.018), older age (OR: 1.02; 95% CI: 1.01 to 1.03; *P* < 0.001), and dialysis vintage (OR: 1.20; 95% CI: 1.13 to 1.29; *P* < 0.001) were significantly associated with mortality (Table 3). Those who were referred from another dialysis facility had a lower mortality (OR: 0.46; 95% CI: 0.34 to 0.64; *P* < 0.001). After adjusting for other factors, only older age (OR: 1.02; 95% CI 1.01 to 1.03; *P* < 0.001) retained significant association with mortality (Table 3).

**Table 3:** Logistic regression analysis showing association of death with clinical variables

Variables	Unadjusted		Adjusted	
	OR (95% CI)	*P*	OR (95% CI)	*P*
Male	1.19 (0.88 to 1.60)	0.262	1.22 (0.88 to 1.69)	0.238
Tobacco use	0.72 (0.45 to 1.15)	0.172	0.73 (0.42 to 1.26)	0.263
Exercise	1.03 (0.69 to 1.54)	0.874	0.85 (0.55 to 1.30)	0.443
Diabetes	1.65 (1.20 to 2.25)	0.002	1.00 (0.68 to 1.45)	0.979
Dyslipidemia	1.61 (0.60 to 4.34)	0.343	0.82 (0.27 to 2.52)	0.729
Heart disease	1.81 (1.11 to 2.95)	0.018	1.37 (0.79 to 2.36)	0.261
Hypertension	1.74 (1.33 to 2.29)	<0.001	1.28 (0.89 to 1.83)	0.179
Age (years)	1.02 (1.01 to 1.03)	<0.001	1.02 (1.01 to 1.03)	<0.001
Dialysis duration, days^a^	1.20 (1.13 to 1.29)	<0.001	1.07 (0.97 to 1.18)	0.199
Referred from another facility	0.46 (0.34 to 0.64)	<0.001	0.72 (0.45 to 1.15)	0.172

CI, confidence interval; OR, odds ratio.

a Duration of dialysis covariate is log transformed for analysis purpose

## Discussion

This is the first systematic report of the impact of COVID-19 on the outcomes of patients on in-center hemodialysis from the developing world. In the absence of a national dialysis registry, this analysis from a large cohort comes closest to a nationwide representation of the health effects of this pandemic in India. We found that the prevalence of infection was 20-fold greater in this population compared to that reported in the general population in India during this period (8.7% and 0.44%, respectively). This is greater than that described in the Renal Epidemiology and Information Network registry data from France (3.3% vs. 0.2%).^1^ In addition to the increase in risk due to repeated contact with the health care system, the higher prevalence could also be contributed by opportunities for more frequent screening and testing. The male predominance likely reflects the male dominance in the general dialysis population. An overwhelming proportion (99%) of COVID-19–positive patients in this cohort was admitted to hospitals in compliance with local policies.

The COVID-19 surge in India followed those in China, Western Europe, and North America. This allowed the Indian centers to adopt the best practices implemented in dialysis centers in those parts of the world. There were unique challenges, however, related to the Indian health care system, such as the closure of units in certain hospitals that were converted into COVID-19 hospitals and other centers turning COVID-19–positive patients away because of lack of resources that produced additional hardships for dialysis patients in India.^5^^,^^6^ Approximately 30% of COVID-1–positive patients had been referred from other dialysis centers.

Approximately one-quarter of all COVID-19–positive dialysis patients died. This mortality rate is comparable to that reported by other studies on hemodialysis patients from high-income countries, despite the Indian dialysis population being younger and having a lower prevalence of comorbidities. The mortality was indeed much greater than the COVID-19 case fatality rate among the general population of India (1.45%). However, the mortality rate among non–COVID-19 patients during the study period in the network was 19.26%, which suggests that the excess mortality in the COVID-19–positive population was only approximately 3.7%. The mortality in the NephroPlus dialysis cohort during the corresponding period in 2019 was 15%, suggesting that the impact of COVID-19 during the study period was not limited to those who were infected with the virus. Our finding confirms the high mortality reported among dialysis patients in general during the pandemic,^5^^,^^7^ attributable to other factors related to the pandemic or the lockdown such as difficulty in transport, closure of dialysis facilities, reduced dialysis frequency, decreased inpatient and outpatient care, and especially financial difficulties.

As expected, elderly males, those with diabetes, hypertension, pre-existing heart disease, and those with longer dialysis vintage were at increased risk of mortality. The mortality risk factors are similar to those reported in other studies among the dialysis population and general population studies from India.

The strength of our study includes nationwide coverage with a large population base that was screened using a uniform protocol, and the completeness of outcome data. We show that despite relatively limited resources, it was possible to implement COVID-19 protocols in dialysis units. This is important because in-center dialysis is the overwhelming dialysis modality during COVID-19 in India, with very low penetration of home dialysis and an almost complete stoppage of transplantation.^8^ There are some limitations, however. Although the network had a uniform temperature- and symptom-screening protocol before unit entry, the implementation might have differed based on local practice adherence. The absence of universal screening might have led to missing out of asymptomatic individuals and an overestimation of case fatality rates.

The protocols for screening during the study period were constrained by government directives, local preferences, access to testing, and self-reporting of symptoms. A small study of COVID-19 dialysis from Mumbai had shown that more than 50% of patients were asymptomatic or had mild disease.^7^ Finally, we did not have data on the severity of COVID-19 infection and treatment protocols in the individuals.

In view of the ongoing surges, the threat by the pandemic will remain significant for this vulnerable population. This can be minimized by liberal testing protocols and persisting with steps intended to minimize disease transmission. The COVID-19 vaccination program started in India on January 16, 2021. Given the high risk of death, especially among the younger population on dialysis, experts have called for prioritization of dialysis patients for vaccination before other high-risk groups such as the obese and smokers and those with heart disease and obesity.^9^ It is worth pointing out that a significant proportion of tobacco users use chewing tobacco, which does not cause lung injury to the same extent as smoking.

To conclude, our study confirms that the in-center dialysis population has a high risk of acquiring COVID-19 infection and has poor outcomes once infected. Our study reinforces the need to implement strict measures targeting personal protection as well as the need to find evidence-based approaches to prevent the development of COVID-19 in this high-risk population.

## Disclosures

SS, MGB, and KDS are employed by NephroPlus. VJ has research grants from Baxter, GSK and reports Consultancy and Advisory Board honoraria from Baxter Healthcare, and AstraZeneca, outside the published work. All other authors reported no conflict.
