# Supplementary material for: Outcome and Determinants of Outcome of COVID-19 Infection Among Hemodialysis Patients: Findings From a National Dialysis Network Program in India
Source: Kidney Int Rep. 2021 Mar 15;6(5):1429–32. doi: 10.1016/j.ekir.2021.03.003 (PMC7956901; doi:10.1016/j.ekir.2021.03.003)
Supplement: Supplementary File (PDF) [file mmc1.pdf]

METHODS

The study was a prospective, observational cohort study in COVID positive dialysis patients identified in 75 centers in 50 cities across India under the NephroPlus dialysis network from 24th March 2020 to 31st December 2020.

Patients were screened for SARS-CoV-2 infection as per the local guidelines. All patients reported to a screening area before entering the dialysis unit. All subjects who were symptomatic or who had come in contact with another SARS-CoV-2 positive individual were tested using RT-PCR. Some patients were referred to a NephroPlus center after being detected positive at another dialysis facility because they did not have the facility to dialyse patients with COVID-19. All those found to be positive were admitted to a designated COVID hospital as per the prevalent policy until October 2020. From November 2020, the decision to admit patients was made on the basis of disease severity - only those with moderate - severe disease where admitted whereas those with mild disease were managed on ambulatory basis

Data including demographic characteristics, and comorbidities were extracted from unit records. Data on outcome was collected by following up patients in their respective hospitals or homes.

Statistical analysis explored the association between comorbidities and demographic characteristics and mortality in COVID positive dialysis population. Descriptive statistics were used to describe the baseline characteristics of study subjects. Data is presented as mean ± Standard deviation and number (percentage). Continuous data were compared using paired *t* tests. Duration of dialysis covariate was log transformed for analysis. Categorical data was expressed as proportions and compared using the Chi-square and Fisher exact test. We used uni- and multi-variate logistic regression models to examine the association between clinical and demographic variables and death. All the analyses were performed using IBM® SPSS® version 26. A two-tailed p value 0.05 was considered significant.
